# Supplementary material for: Evaluation of Pre-Pandemic Trivalent COBRA HA Vaccine in Mice Pre-Immune to Historical H1N1 and H3N2 Influenza Viruses
Source: Viruses. 2023 Jan 11;15(1):203. doi: 10.3390/v15010203 (PMC9861495; doi:10.3390/v15010203)
Supplement: Supplementary file 1 [file viruses-15-00203-s001.zip › viruses-2126212-supplementary.pdf]

Evaluation of pre-pandemic trivalent COBRA HA vaccine in mice pre-immune to historical H1N1 and H3N2 influenza viruses

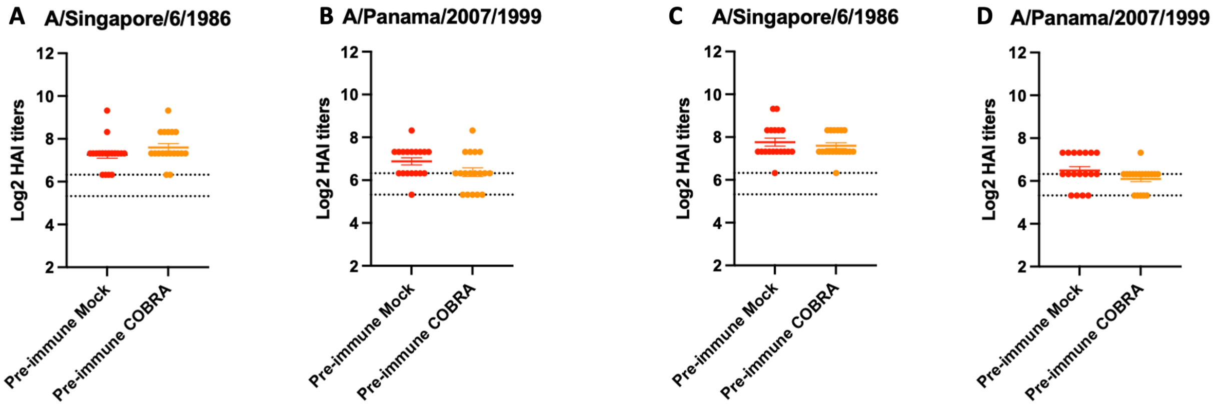

Figure S1 Pre-immune mice seroconversion to H1N1 and H3N2 strains. HAI titers were tested on the serum collected 14 days after mice intranasally inoculated with equal concentrations of A/Singapore/6/1986/H1N1 and A/Panama/2007/1999/H3N2 viruses. **A,B** are results of mice in Pre-immune Mock and Pre-immune COBRA groups later being challenged with A/Mallard/Minnesota/2008/H2N3 virus. **C,D** are results of mice in Pre-immune Mock and Pre-immune COBRA groups later being challenged with A/Sichuan/26221/2014/H5N6. **A,C** are HAI titers against A/Singapore/6/1986/H1N1. **B,D** are HAI titers against A/Panama/2007/1999/H3N2. Top and bottom dash lines represents HAI of 1:80 and 1:40. N= 18 per group in each experiment.

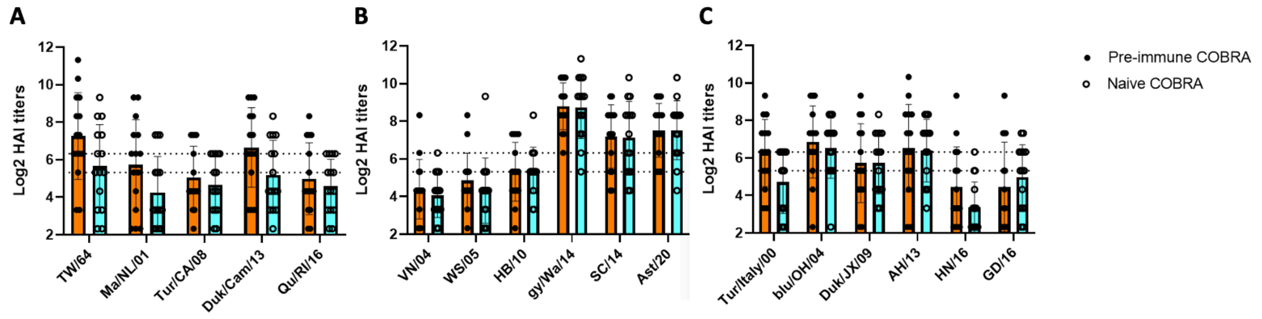

Figure S2 H1/ H3 pre-immunity does not affect H2/H5/H7 HAI titers after two COBRA rHA vaccinations. HAI activities from both COBRA vaccinated groups were compared. H2 (**A**), H5 (**B**), H7 (**C**) HAI titers are shown. Solid dots in orange bars represent mice in Pre-immune COBRA group, hollow dots in blue bars represent mice in Naïve COBRA group. N= 15 per group in each group.

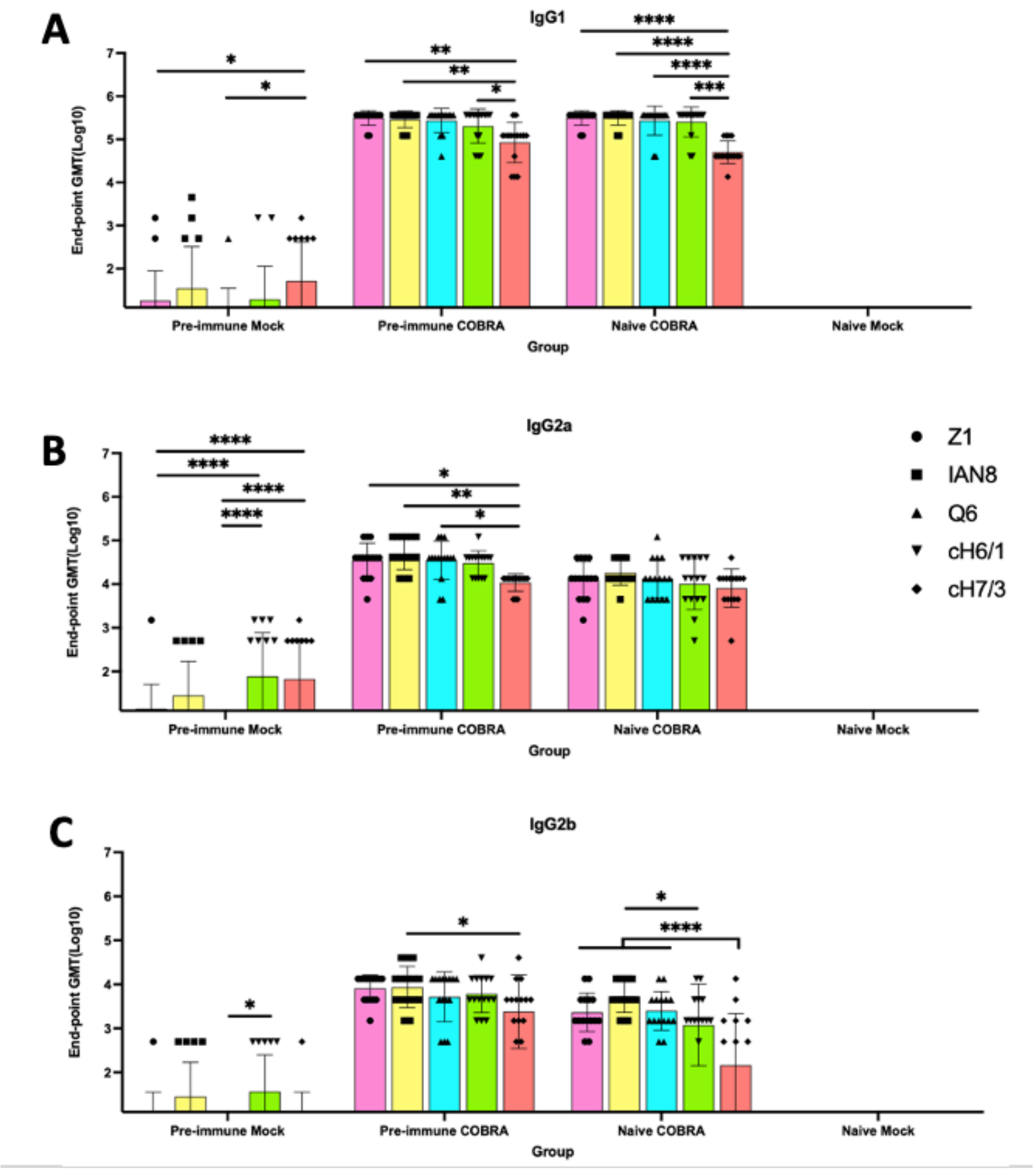

Figure S3 COBRA HA vaccinations elicit high IgG1, IgG2a and IgG2b. Serum from DBA2/J mice collected 2 and 3 weeks after boost vaccination was used to determine anti-Z1, anti-IAN8, anti-Q6, anti-cH6/1, anti-cH7/3 endpoint titer in each group. IgG1 (**A**), IgG2a (**B**), IgG2b (**C**) endpoint titers are shown. Data shown is mean ± SEM, n= 15 per group in each experiment; *P<0.05, **P<0.01, ***P<0.001, ****P<0.0001 (two-way ANOVA with Turkey's post-test in panels A, B, C).

Group	Mouse ID	D7 BW % in H2 challenge	H2 (≥1:40)	H5 (≥1:40)	H7 (≥1:40)	total
Pre-immune COBRA	61	99.61%	3/5	5/6	4/6	12/17
	62	99.20%	1/5	4/6	4/6	9/17
	63	93.66%	5/5	2/6	6/6	13/17
	64	94.61%	2/5	6/6	1/6	9/17
	65	100.75%	5/5	4/6	5/6	14/17
	66	97.10%	2/5	3/6	1/6	6/17
Naïve COBRA	91	91.38%	2/5	5/6	4/6	11/17
	92	89.33%	3/5	4/6	3/6	10/17
	93	98.06%	0/5	3/6	4/6	7/17
	94	87.80%	2/5	5/6	4/6	11/17
	95	95.49%	1/5	5/6	4/6	10/17
	96	100%	4/5	5/6	5/6	14/17
**Group**	**Mouse ID**	**D7 BW % in H5 challenge**	**H2 (≥1:40)**	**H5 (≥1:40)**	**H7 (≥1:40)**	**total**
Pre-immune COBRA	181	99.25%	3/5	4/6	4/6	11/17
	182	94.07%	1/5	5/6	4/6	10/17
	183	90.66%	5/5	2/6	6/6	13/17
	184	95.08%	2/5	6/6	1/6	9/17
	185	102.22%	5/5	4/6	5/6	14/17
	186	100%	2/5	3/6	1/6	6/17
Naïve COBRA	211	92.78%	2/5	5/6	4/6	11/17
	212	104.23%	3/5	4/6	3/6	10/17
	213	92.08%	0/5	3/6	4/6	7/17
	214	100%	2/5	5/6	4/6	11/17
	215	91.59%	1/5	5/6	4/6	10/17
	216	99.36%	4/5	5/6	5/6	14/17

Table S1 Body weight change and number of strains be recognized in each HAI panel of mice in both Pre-immune COBRA and Naïve COBRA groups. Top and bottom sections are mice in H2 and H5 challenge, respectively. N=5 in H2 HAI panel, N=6 in H5 HAI panel, N=6 in H7 HAI panel, total N=17.
